# Machine translation: Turkish–English bilingual speakers’ accuracy detection of evidentiality and preference of MT

**DOI:** 10.1186/s41235-024-00535-z

**Published:** 2024-02-16

**Authors:** Sümeyra Tosun

**Affiliations:** Department of Psychology, Medgar Evers College, CUNY, New York, NY USA

## Abstract

Machine translation (MT) is the automated process of translating text between different languages, encompassing a wide range of language pairs. This study focuses on non-professional bilingual speakers of Turkish and English, aiming to assess their ability to discern accuracy in machine translations and their preferences regarding MT. A particular emphasis is placed on the linguistically subtle yet semantically meaningful concept of evidentiality. In this experimental investigation, 36 Turkish–English bilinguals, comprising both early and late bilinguals, were presented with simple declarative sentences. These sentences varied in their evidential meaning, distinguishing between firsthand and non-firsthand evidence. The participants were then provided with MT of these sentences in both translation directions (Turkish to English and English to Turkish) and asked to identify the accuracy of these translations. Additionally, participants were queried about their preference for MT in four crucial domains: medical, legal, academic, and daily contexts. The findings of this study indicated that late bilinguals exhibited a superior ability to detect translation accuracy, particularly in the case of firsthand evidence translations, compared to their early bilingual counterparts. Concerning the preference for MT, age of acquisition and the accuracy detection of non-firsthand sentence translations emerged as significant predictors.

## Introduction

Machine translation (MT) is the process of using automated software to translate text from one language to another, catering to a wide array of language pairs. While MT has been in existence since the 1950s, it experienced a significant transformation in the past two decades, shifting from rule-based MT to statistical MT and more recently to neural machine translation (NMT). This evolution, especially the advent of deep learning, has dramatically enhanced the quality of MT (Burchardt et al, 2017; Melby, 2020; Popović, 2017; Turovsky et al., 2022). Some scholars argue that recent improvements have elevated MT quality to a level comparable to human translation (HT) for specific texts and language pairs (Hassan et al., 2018; Perrault et al., 2019). Nevertheless, there remains a lack of consensus regarding the validity of this assertion and the criteria used to evaluate MT quality (Pym, 2020; Toral et al., 2019). Even so, it is widely acknowledged that MT technology has made remarkable progress recently although these advancements vary across language pairs, with popularity influencing the level of investment in data acquisition (Perrault et al., 2019).

As neural MT continues to advance, it is likely that MT will become an even more prevalent technology, necessitating engagement from professional translators. This progress places MT at the forefront of Artificial Intelligence-Mediated Communication (AI-MC), characterized as “interpersonal communication that is not merely transmitted by technology but is modified, augmented, or generated by a computational agent to achieve communication goals” (Hancock et al., 2020, p. 90). Recent research on interactions with MT has shown that its usage does not hinder the translation process or necessarily impact the final quality of the translated text (e.g., Gaspari et al., 2014; Koponen, 2012; Moorkens, et al., 2015; Teixeira, 2014). However, professional translators still exhibit substantial resistance to adopting MT as an aid and notice more errors in MT. This study aims to explore whether the preferences of non-professional Turkish–English bilingual speakers for MT and their ability to detect translation errors are associated.

Modern technologies are increasingly deployed to surmount language barriers, extending their reach beyond personal use into critical domains like healthcare, legal proceedings, and law enforcement. The accessibility and societal influence of machine translation (MT) are currently under scrutiny, with a focus on ensuring the inclusive participation of diverse social groups in communication processes (Vieira et al., 2021). The proliferation of freely accessible MT systems online has democratized language translation, bridging gaps in both developed and developing nations. Google Translate, for instance, processes an astounding 150 billion words daily, highlighting the far-reaching societal implications of MT beyond the translation community (Davenport, 2018). However, there is a recognized gap in public awareness regarding the capabilities and quality of machine translation (Kasperé et al., 2021).

While MT is rapidly dismantling language barriers and closing in on human-level translation accuracy and efficiency, human intervention remains crucial to mitigate the potential negative consequences of its use in society (Hoi, 2020). Effective communication processes supported by MT can attain high quality, provided that participants are cognizant of the technology's limitations (Yasuoka & Bjorn, 2011). Furthermore, research has shown that MT can play a pivotal role in reducing the exclusion of ethnic minorities across various fields (Taylor et al., 2015).

### Recent research on MT

Extensive research has investigated the quality of machine translation and the utilization of post-editing techniques (Nurminen & Koponen, 2020; Ortega et al., 2019; Rossi & Carré, 2022; Ueffing, 2018; Vardaro et al., 2019, among others). Various studies employing diverse research methodologies have recognized the advantages of post-editing machine translation across different language pairs (Carl et al., 2011, 2015; Moorkens, 2018; Stasimioti & Sosoni, 2021). Additionally, investigations have addressed the acceptability of machine-translated content (Castilho, 2016; Castilho & O’Brien, 2017; Rivera-Trigueros, 2021; Taivalkoski-Shilov et al., 2022). However, these studies have primarily focused on the attitudes and perceptions of translation students, novice translators, professional translators, and post-editors, likely due to the convenience of access to respondents and research design (Moorkens et al., 2015; Rossi & Chevrot, 2019; Ferreiraa et al., 2021). Surprisingly, the acceptability of machine-translated content among non-professional users remains relatively understudied.

Prior research in machine translation has predominantly focused on professional translators' viewpoints, including their perception of MT as a potential industry threat (Vieira, 2020), the factors influencing their adoption or rejection of MT (Cadwell et al., 2018), and the impact of MT advancements on their practices, self-perception, and professional standing (Läubli & Orrego-Carmona, 2017; Sakamoto, 2019). These studies have consistently highlighted translators' concerns and their generally lower regard for MT regarding quality and utility. However, it is worth noting that some of this resistance diminishes when translators are involved in the development and implementation of MT software (Rossi & Chevrot, 2019). As MT increasingly permeates non-translator usage in various social communication contexts, focusing solely on professional translators' attitudes limits our understanding of the broader societal impacts of this technology. Therefore, it is essential to consider both translator and non-translator populations when exploring public perceptions of MT, particularly from ordinary users' perspectives, given the various purposes for which they casually employ machine translation in their daily lives (Kasperé et al., 2021).

The studies, particularly on professional translators, demonstrate that machine-translated text typically demands a higher cognitive load, to varying degrees, compared to human-translated or post-edited content. Earlier user-centered research analyzed raw machine translation, and identified reduced usability of machine-translated instructions compared to post-edited output (Carl et al., 2011; Castilho, 2016; Castilho et al., 2014; Doherty & O’Brien, 2014; Doherty, *2016*; Daems et al., 2017; Ferreiraa et al., 2021; Guerberof Arenas et al., 2021; Hu et al., 2020; Jakobsen & Jensen, 2008; Kasperé et al., 2023; Moorkens, 2018; Stasimioti & Sosoni, 2021; Vardaro et al., 2019).

Similarly, studies on non-professional users, that have examined the acceptability of machine-translated text, revealed that readers spend more time and cognitive effort on machine-translation errors compared to correct segments of text (Colman et al., 2021; Kasperavičienė et al., 2020). The presence of errors in machine-translated segments increased the cognitive processing demands. Hence, post-editing machine-generated translations remains crucial to ensure precise language translations (Gaspari et al., 2014; Macías et al., 2020; Taylor et al., 2015).

Other studies have investigated how non-professionals or individuals with low proficiency perceive the acceptability of machine-translated texts in various languages. The individuals utilize machine translation for various purposes, often without a full understanding of how it works or the quality it provides. For instance, in a survey of 400 participants, the acceptability of machine-translated text from English to Lithuanian was influenced by factors like age and education. Less educated and older participants were more inclined to view machine translation as reliable and satisfactory (Kasperé et al., 2021). However, when the situation is ethically charged (e.g., in legal settings) both translators and non-translators exhibit a negative bias toward MT (Asscher & Glikson, 2023).

In a study by Rossetti et al. (2020), 61 participants assessed the “impact of machine translation and post-editing awareness” on comprehension and trust when reading crisis messages in English and Italian. The results showed no significant differences in comprehension and trust between raw machine-translated and post-edited text. However, participants with limited English proficiency had a more favorable view of raw machine-translated text in terms of comprehension and trust. Another study involving translation agencies, professional translators, and clients/users of professional translation investigated user awareness of machine translation through surveys (García, 2010). The study focused on the acceptability and evaluation of machine translation from Chinese into English. The findings indicated that less than 5% of professional translators considered the quality of machine translation to be very high. Translation agencies shared a similar view with the translators. Among clients/users of professional translation (about 30%) who were aware of and requested machine translation, there was an intermediate or positive assessment of its quality.

For closely related languages in MT, a lexical analysis aided by translation rules often suffices, omitting the need for extensive semantic analysis. Rule sets for translation are more manageable in close language pairs compared to unrelated ones, simplifying the rule coding process (Altintas & Cicekli, 2022). NMT systems utilize typological similarities among languages to create clusters (Tan et al., 2019). Typologically distinct languages require more effort in NMT and might reveal fewer satisfying results. Thus, the current study, following an offline methodology, focused on a typologically distinct language pair, namely Turkish and English, and their non-professional bilingual speakers. It aimed to determine their ability to detect accuracy in machine translation and their preference for MT in need of a translator, particularly concerning the linguistically subtle yet semantically significant concept of evidentiality.

### Evidentiality

Evidentiality, a linguistic concept, relates to how a language conveys the source of knowledge regarding an event described (Aikhenvald, 2004). It allows speakers to indicate whether they personally witnessed an event or acquired information about it from others. In certain languages like Turkish, evidentiality is grammatically marked, requiring speakers to specify their information source when describing past events. In Turkish, firsthand experiences are marked with “-di,” while non-firsthand information, such as hearsay or inference, is marked with “-miş.” In contrast, languages like English offer optional means to express evidential-type meanings, relying on lexical or constructional choices rather than grammatical markers. This flexibility allows English speakers to decide whether to incorporate evidentiality in their descriptions, using words like “*apparently*” or “*seemed like*” to indicate their source of information. While evidentiality is not grammatically obligatory in English, it can still be expressed. However, due to its optional nature, it is less likely to be consistently and frequently expressed compared to Turkish, where it is grammatically mandatory. Translating from English to Turkish is relatively straightforward because there is a single marker for different sources of non-firsthand knowledge. However, challenges arise when translating from Turkish, which has one marker for non-firsthand information, to English, which offers multiple options (Filipović, 2017).

Linguistic and psycholinguistic research highlights that apart from indicating the source of knowledge, evidentiality communicates the epistemic value of information (Aikhenvald, 2004; Aksu-Koç, 2016; Arslan, 2020; Plungian, 2001; Tosun & Vaid, 2018; Willett, 1988). A proposition expressed with a firsthand source signifies higher confidence in its occurrence (higher epistemic value), while non-firsthand statements suggest less certainty, casting doubt on the proposition's actual occurrence (lower epistemic value).

Monolingual Turkish speakers typically acquire evidentiality in early childhood (Aksu-Koç et al., 2009). Research by Aksu-Koç and colleagues demonstrates that Turkish-speaking children start using evidential markers between 18 months and 3 years of age. However, some studies have indicated that comprehending sentences with evidential marking may be challenging until around the age of 3 (Öztürk & Papafragou, 2016). Bilingual speakers’ acquisition of evidentiality demonstrated differences from monolingual speakers. It is noteworthy that some bilingual speakers may not fully acquire evidentiality. Heritage speakers of Turkish, who are fluent in Turkish but learned it as a heritage language, have been found to have lower sensitivity to evidentiality markers compared to native Turkish speakers (Arslan, 2020; Arslan & Bastiaanse, 2020; Arslan et al., 2017; Schmid & Karayayla, 2020). Similarly, second-language learners of Turkish struggle with the various uses of non-firsthand evidential markers (Kaya-Soykan et al., 2023). Studies involving English speakers learning Japanese, a language that grammatically encodes evidentiality, have also shown that learners use evidential markers less frequently and correctly compared to native speakers (Ishida, 2006; Narita, 2011).

A recent study investigating the typological differences in evidentiality examined Turkish–English bilinguals in a bilingual setting (Tosun & Filipović, 2022). This study, similar to previous research, found that the AoA influenced bilingual speakers’ performance in a bilingual context. Early bilinguals were typically heritage speakers of Turkish and their parents were born in Turkey and residing in the USA or the UK. Late bilinguals were immigrants from Turkey currently living in the USA or the UK. Participants were presented with sentences in both English and Turkish containing information marked as firsthand or non-firsthand. They were tasked with translating these sentences between the two languages and making judgments about the likelihood of the described events actually occurring. The results revealed that both groups performed similarly when translating firsthand-marked sentences. However, late bilingual speakers noticed non-firsthand sources when translating from English to Turkish, while early bilingual speakers tended to ignore information about non-firsthand sources when translating from Turkish to English. Additionally, late bilinguals were more likely to consider events described in firsthand-marked sentences as having actually occurred, compared to those described in non-firsthand sentences. These findings highlight the typological differences between English and Turkish and the challenges faced by early bilinguals in accurately discerning information sources. The study prompts questions about how accurately they can detect machine translation errors and how likely they are to prefer machine translation.

### Present study

This study delved into the accuracy of Turkish–English bilingual speakers in detecting machine translation (MT) errors in translations of past events. The central focus was on evidentiality, a key linguistic property, given its distinctive structures in these two languages. The primary aim of the study was to explore how proficient MT users were in recognizing errors within MT outputs (rather than investigating the quality of MT outputs) and how this proficiency was associated with their use and preference for MT tools. The research aimed to address several key questions:

1. How proficiently can bilingual speakers identify inaccuracies of evidentiality in MT of past events?
2. Does their ability to discern differences in evidentiality in translations predict their preference for MT tools in need of a translator?
3. Do specific contexts, where MT is applied, such as medical, legal, academic, or daily communication settings, influence their MT preference judgments?
4. Does the AoA of bilingual individuals impact their preference for MT and their accuracy in detecting translation errors?

Given the escalating use of MT in our interconnected world, gaining insights into bilingual speakers' attitudes toward MT holds substantial significance, particularly in contexts where discourse plays a pivotal role in decision-making processes.

## Method

### Participants

A total of 36^1^ participants (all female^2^) were recruited in the USA and South Africa. AoA threshold was established at 12 years old. Participants who acquired their second language (English) after the age of 12 were categorized as late bilinguals. The late L2 English bilingual group consisted of 15 participants, with a mean age of 33 (*SD* = 10.38). Their mother tongue was Turkish, and it was acquired in Turkey. They immigrated to the US or South Africa for educational or economic purposes, where they acquired their L2 English. The L2 English was rated as worse than their Turkish by 34% and the remaining stated that their English was as good as their Turkish in this group. They graded their Turkish proficiency a 7 and English proficiency a 6 out of a 7-point scale. They indicated their use of Turkish as 60% and English as 40% daily (*SD* = 6.12). The early bilingual group consisted of 21 participants with a mean age of 17.57 (*SD* = 3.63). They were generally heritage learners who learned and spoke Turkish at home and learned and spoke English at school or outside of home. All early bilingual participants stated that their English was better than their Turkish. Out of a 7-point scale, they judged their Turkish proficiency as 5 (including reading and writing) and English proficiency as 7. Early bilinguals also indicated that approximately 78% of the time, they used English daily (*SD* = 8.56).

### Materials and measures

For the translation accuracy detection phase, three variables were manipulated within subjects: Translation direction (English to Turkish vs Turkish to English), Source of information (Firsthand vs Non-firsthand), and Translation accuracy (Correct vs Incorrect). Thus, there were 8 total conditions. A total of 80 sentences were divided evenly between these 8 conditions, which made 10 sentences per condition.

Half of the sentences were presented as the source language was Turkish and the target language was English, while the other half were given the other way around. For half of the Turkish sentences (20 sentences), the past tense suffix used was the firsthand form (-*di: Suna eski kocasını affetti*) and for the remainder (20 sentences) the non-firsthand past tense suffix was used (*-miş: Suna eski kocasını affetmiş*). For half of each form, the English translations (10 sentences) were presented correctly (with a phrase added to indicate the source of information for non-firsthand sentences such as *apparently*, *it seemed like*) and the other half translations were presented incorrectly (10 sentences). Like Turkish to English stimuli, half of the sentences (20 sentences) of English to Turkish translations were introduced in firsthand form (e.g., *Sue forgave her ex-husband*). The other half (20 sentences) was in non-firsthand form and contained one of the following expressions that signal evidential meanings (and that are most often used as translation equivalents for the Turkish non-firsthand evidential): it appeared, it seemed, must have, it looked like, and apparently (e.g., *It seemed Sue forgave her ex-husband)*. Half of the English-to-Turkish translation sentences were presented in the correct translation format (where the source of information was translated accurately) and the other half were presented in an incorrect translation format. Stimuli were presented in the blocks of the direction of translation and the direction of translation blocks were counterbalanced. Per language, firsthand and non-firsthand sentences were presented in a fixed random order. Also, for each language, the particular sentences chosen to be in firsthand versus non-firsthand form and correct versus incorrect translation form were counterbalanced across participants.

The sentences were simple declarative, transitive sentences, each containing a verb in the past tense. All sentences were roughly of similar length and were adapted from stimuli created by Tosun and Filipović (2022). While the original sentences were translated using Google Translate, the author later manipulated them to create the above-mentioned conditions. This involved adding or removing evidential expressions in English and modifying the Turkish suffixes. Expressions such as “it seemed like,” “apparently,” “must have,” and “it looked like” were used for non-firsthand sentences. These phrases were positioned either at the start of the sentence or just before the verb, depending on their natural usage in English.

For this section, participants’ *hit rates* (total number of detections of translation accuracy correctly) and *false alarms* (judging the incorrect translations as correct) for each source were computed. The false alarm term was entered into the accuracy detection computation to achieve a more balanced and fine-tuned evaluation of detection performance. Finally, translation accuracy detection was calculated as the difference between hits and false alarms. Thus, the dependent variable presented the pure ability to accurately discriminate between correct detection and noise while minimizing false identifications. This increased the sensitivity and discriminability of the correct detection measure.

A language background questionnaire was utilized to detect the bilingual participants’ language history, their AoA, and other relevant metadata such as frequency of daily use of each language and proficiency self-assessment. MT use, trust, and preference were measured by employing a questionnaire. In this questionnaire, participants were asked about their usage patterns of MT tools (How frequently do you use the machine translation tools on a daily basis? Please use the slider to adjust your frequency use from 0 to 100), and the level of trust they place in these tools (How much do you trust the accuracy of the translation that is produced by the machine translation tools? Please use the slider to adjust your trust rate from 0 to 100). Subsequently, they were asked about their likelihood of preferring MT in various settings (How much would you prefer to be translated by MT in need of a translator in the following settings? Please use the slider to adjust your preference level from 0 to 100), including medical, legal, academic, and daily contexts.

### Procedure, design, and data analysis

The experiment was conducted through Qualtrics. Participants received the language background questionnaire first followed by the MT use and preference questionnaire. Then they were instructed that they would see some sentences followed by their equivalent translation completed by a machine translation tool. The sentences were presented side by side and without time limitation. They were asked to read each sentence and its translation carefully, and then make a judgment about whether it was translated accurately. The sentences (both the original sentence and the translation) were available on the screen while participants were making their judgments and indicating their responses. They were additionally asked to indicate the kind of inaccuracy if they thought it was not accurately translated. The order of the translation direction (Turkish to English and English to Turkish) was counterbalanced. The experiment took approximately forty-five minutes to complete, and participants received compensation when they submitted their completed forms.

#### Translation accuracy detection

This portion of the study had a 2 (AoA: Early vs. Late L2 English) × 2 (Direction: Turkish to English (T to E) vs. English to Turkish (E to T)) × 2 (Source: Firsthand vs. Non-firsthand) mixed design. Group was the between-subjects variable, and other variables were manipulated as within subjects. The dependent variable was participants’ translation accuracy detection, which was calculated as the difference between hits and false alarms. A 2 × 2 × 2 repeated measures ANOVA was conducted and the Bonferroni method was used for post-hoc comparisons.

#### MT preference

In this analysis, the possible predictors of MT preference in need of a translator were questioned. A series of multiple regression analyses were employed in which the MT preference was the dependent variable and MT use frequency, trust in MT, translation accuracy detection scores of non-firsthand sources^3^ (both T to E and E to T), and AoA were entered as the predictors. No interaction terms were entered into the model. The analysis was conducted separately for the preference score of each setting (Medical, Legal, Academic, and Daily).

## Results

Before presenting further analysis the early and late bilingual participants’ frequency of MT use and trust in MT were analyzed. An independent sample t-test was utilized to examine the difference between the two bilingual groups. The results revealed that late bilinguals (*M* = 26.8, *SD* = 15.9) used MT more frequently than early bilinguals (*M* = 16.4, *SD* = 10.9, *t* (45) = 2.64, *p* = 0.01, Cohen’s d = 0.78). However, their trust in MT did not reveal a significant difference (*M*_*Late*_ = 66.3, *SD*_*Late*_ = 28.6; *M*_*Early*_ = 63.2, *SD*_*Early*_ = 20.3, *t* (45) = 0.43, *p* = 0.67, Cohen’s d = 0.13).

### Translation accuracy detection

The summary of descriptive statistics is presented in Table 1. The results revealed a significant main effect of Source of information [*F* (1, 34) = 61.64, *p* < 0.001, *η*_*p*_^*2*^ = 0.65]. Firsthand source’s translation accuracy was detected better than non-firsthand source (Mean Difference (*MD*) = 0.43). Source by AoA interaction was significant [*F* (1, 34) = 18.16, *p* < 0.001, *η*_*p*_^*2*^ = 0.35]. The late bilingual group was significantly better in the detection of firsthand translation accuracy than early bilinguals (*MD* = 0.43, *p* < 0.001) although, the difference disappeared in non-firsthand translations (*MD* =  − 0.03, *p* = 1). Within late bilinguals, firsthand translation accuracy was better detected than non-firsthand translation (*MD* = 0.66, *p* < 0.001). The same effect appeared in the early bilingual group, although the difference was relatively smaller (*MD* = 0.20, *p* = 0.05).

**Table 1:** Summary of accuracy detection of MT across AoA, source of information and translation direction

	Firsthand				Non-firsthand			
	Late		Early		Late		Early	
	T to E	E to T	T to E	E to T	T to E	E to T	T to E	E to T
Mean	0.95	0.55	0.46	0.18	− 0.22	0.40	0.00	0.24
SE	0.08	0.10	0.07	0.08	0.15	0.09	0.13	0.07

Direction by Source interaction was also significant [*F* (1, 34) = 47.6, *p* < 0.001, *η*_*p*_^*2*^ = 0.58]. T to E firsthand translations were detected significantly better than non-firsthand translations in the same direction (*MD* = 0.81, *p* < 0.001) although, the difference disappeared in E to T translations (*MD* = 0.05, *p* = 1). Within firsthand translations, T to E translation accuracy was better detected than E to T translation (*MD* = 0.33, *p* < 0.001). The reverse effect was found in non-firsthand sentences, where T to E translations were less accurately detected than E to T translations (*MD* =  − 0.43, *p* < 0.001).

**Fig. 1:**
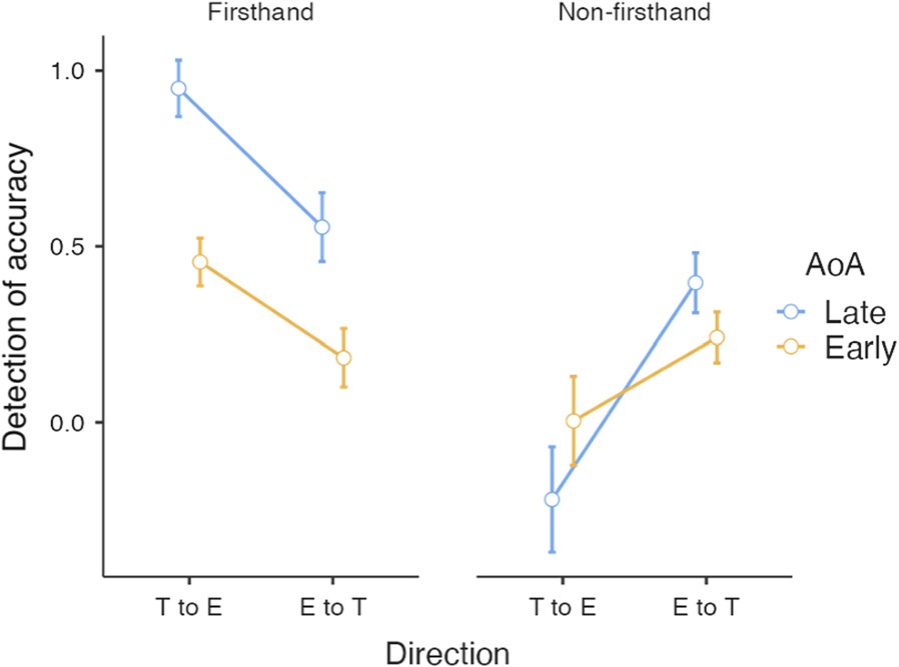
This figure depicts the three-way interaction of Direction by Source by AoA

Finally, the three-way interaction (see Fig. 1), Source by AoA by Direction was significant [*F* (1, 34) = 5.19, *p* = 0.03, *η*_*p*_^*2*^ = 0.13]. When translations were from T to E, late bilinguals detected firsthand sentences more accurately than early bilinguals (*MD* = 0.49, *p* = 0.001), although, the difference disappeared when translations were from E to T (*MD* = 0.37, *p* = 0.18). The direction effect also disappeared for early and late bilinguals in non-firsthand translations (T to E: *MD* =  − 0.22, *p* = 1; E to T: *MD* = 0.15, *p* = 1). Within late bilinguals, T to E firsthand translations were more accurately detected than T to E non-firsthand translations (*MD* = 1.17, *p* < 0.001) and E to T firsthand translations (*MD* = 0.39, *p* = 0.012). Non-firsthand translation accuracy demonstrated the otherwise, T to E translations were less accurately detected by late bilinguals than E to T translations (*MD* = 0.61, *p* < 0.001).

Within early bilinguals, T to E firsthand translations were detected more accurately than non-firsthand translations (*MD* = 0.45, *p* = 0.05), although they were equally better detected as E to T firsthand translations (*MD* = 0.27, *p* = 0.09). Further there was no difference between non-firsthand sentences of both directions (*MD* =  − 0.23, *p* = 0.1) for early bilinguals.

### MT preference

#### The effect of settings

MT preference of four settings was compared by utilizing a repeated measure ANOVA as entering AoA as a between-subject factor. The results revealed a significant Settings main effect [*F* (3, 126) = 4.09, *p* = 0.008, *η*_*p*_^*2*^ = 0.09] and a significant Settings by AoA interaction [*F* (3, 126) = 6.03, *p* < 0.001, *η*_*p*_^*2*^ = 0.13]. Late bilingual speakers preferred to be translated by MT tools in all four settings roughly the same. Early bilingual speakers, on the other hand, demonstrated differences in some settings, in which, they preferred to be translated by MT tools in academic settings more likely than medical (*MD* = 21.5, *p* = 0.06) legal (*MD* = 22.54, *p* = 0.03) and daily (*MD* = 36.92, *p* < 0.001) settings (see Table 2).

**Table 2:** Summary of MT preference of bilingual speakers by AoA and settings

	Medical		Legal		Academic		Daily	
	Late	Early	Late	Early	Late	Early	Late	Early
Mean	52.1	42.8	60.3	41.8	49	64.3	51.5	27.4
SE	7.21	6.58	6.43	5.87	4.91	4.48	6.58	6.01

#### Predictors of MT preference

The summary of the results is presented in Table 3. Four multiple regression analyses were conducted to examine the predictors of MT preferences in Medical, Legal, Academic, and Daily settings. The two non-firsthand source accuracy detection scores (T to E and E to T), MT use frequency, trust in MT, and AoA were entered as predictors.

**Table 3:** Summary of the regression analysis: possible predictors of MT preference in various settings

Model summary	R^2^	F	*p*	ß	*p*
Medical	0.65	11.40	< 0.001		
Frequency of use				− 0.19	0.12
Trust to AI				0.26	0.09
T to E Non-Firsthand				1.62	< 0 .001
E to T Non-Firsthand				− 1.05	< 0 .001
AoA				− 1.39	< 0 .001
Legal	0.68	13.00	< 0.001		
Frequency of use				− 0.23	0.05
Trust to AI				0.29	0.05
T to E Non-Firsthand				1.53	< 0.001
E to T Non-Firsthand				− 1.07	< 0.001
AoA				− 1.80	< 0.001
Academic	0.50	5.94	< 0.001		
Frequency of use				0.19	0.19
Trust to AI				− 0.37	0.05
T to E Non-Firsthand				0.59	0.04
E to T Non-Firsthand				− 1.07	< 0 .001
AoA				− 0.45	0.18
Daily	0.75	18.00	< 0.001		
Frequency of use				0.30	0.01
Trust to AI				− 0.71	< 0 .001
T to E Non-Firsthand				− 0.35	0.08
E to T Non-Firsthand				0.16	0.34
AoA				− 0.99	< 0.001

*Medical settings* The results indicated that there was a collective significant effect among all five predictors (*F* (5, 30) = 11.4, *p* < 0.001, *R*^*2*^ = 0.65). The individual predictors were examined further and indicated that T to E non-firsthand (*B* = 1.62, *t* (30) = 7.06, *p* < 0.001), E to T non-firsthand (*B* =  − 1.05, *t* (30) = 5.39, *p* < 0.001), and AoA (*B* =  − 1.39, *t* (30) = 5.15, *p* < 0.001) were significant predictors. Participants’ accuracy detections of T to E non-firsthand translations had positively predicted their preference for MT in medical settings. The more accurately they could detect the translations from T to E the more likely they preferred MT. However, their accuracy detections of E to T non-firsthand translations were negatively related to their preference. The more accurately they could detect the translations of non-firsthand sentences the less likely they preferred MT in medical settings. AoA was another significant predictor revealing that late bilinguals were more likely to prefer MT than early bilinguals. Finally, the MT use frequency and trust in MT were not significant predictors in the model.

*Legal settings* The results demonstrated a collective significant effect among all five predictors (*F* (5, 30) = 13, *p* < 0.001, *R*^*2*^ = 0.68). The individual predictors were examined further and indicated that T to E non-firsthand (*B* = 1.53, *t* (30) = 6.99, *p* < 0.001), E to T non-firsthand (*B* =  − 1.07, *t* (30) = 5.7, *p* < 0.001), MT use frequency (*B* =  − 0.23, *t* (30) = 2.08, *p* = 0.05), trust in MT (*B* = 0.29, *t* (30) = 2.01, *p* = 0.05) and AoA (*B* =  − 1.8, *t* (30) = 6.99, *p* < 0.001) were significant predictors. Participants’ accuracy detections of non-firsthand sentences in both directions were significant predictors although in opposite directions. T to E non-firsthand accuracy detection had positively predicted their preference for MT in legal settings. The more accurately they could detect the translations from T to E the more likely they preferred MT. However, their accuracy detections of E to T non-firsthand translations were negatively related to their preference. The more accurately they could detect the translations of non-firsthand sentences the less likely they preferred MT in legal settings. The frequency of MT use was negatively related to preference for MT in legal settings where the more frequently participants use MT the less likely they preferred MT as their translator. As expected, trust in MT is a positively related predictor, the more participants trust in MT the more likely they preferred MT. Finally, the AoA effect was similar to the medical setting in which late bilinguals were more likely to prefer MT than early bilinguals.

*Academic settings* The results showed a collective significant effect among all five predictors (*F* (5, 30) = 5.94, *p* < 0.001, *R*^*2*^ = 0.5). Similar to the other two settings, T to E non-firsthand accuracy detection (*B* = 0.59, *t* (30) = 2.13, *p* = 0.04), E to T non-firsthand accuracy (*B* =  − 1.07, *t* (30) = 4.54, *p* < 0.001) were revealed as significant predictors. Additionally, trust in MT (*B* =  − 0.36, *t* (30) = 2.03, *p* = 0.05) was another significant predictor. As participants’ T to E non-firsthand accuracy detection increased their preference of MT increased. As opposed to T to E, as their E to T non-firsthand accuracy detection increased their preference for MT decreased. Further, as participants indicated less trust in MT, they more likely preferred MT. Differently than the other settings AoA was not a significant predictor of MT preference in academic settings along with the frequency of use.

*Daily settings* The results demonstrated a collective significant effect among all five predictors (*F* (5, 30) = 18, *p* < 0.001, *R*^*2*^ = 0.75). The individual predictors were examined further. Differently than the other settings, the translation accuracy detection of non-firsthand sources in both directions were not significant predictors of participants’ preference for MT in daily settings. The frequency of MT use (*B* = 0.3, *t* (30) = 3.06, *p* = 0.005), trust in MT (*B* =  − 0.71, *t* (30) = 5.58, *p* < 0.001) and AoA (*B* =  − 0.99, *t* (30) = 4.33, *p* < 0.001) were significant predictors. The more frequently participants used MT the more likely they preferred to use MT in daily settings. However, the more they trusted in MT the less likely they preferred it in daily settings. The AoA effect was similar to the other setting in which late bilinguals were more likely to prefer MT than early bilinguals.

## Discussion

This study focused on assessing the MT accuracy detection capabilities of Turkish–English bilingual speakers, particularly in the context of translating past events, and examined their MT preferences. The choice of past event translations was driven by a typological difference between Turkish and English, specifically related to evidentiality—a structural variation in indicating the source of information about past occurrences. Despite its subtle structural nature, this distinction had significant semantic implications (e.g., Aikhenvald, 2004; Aksu-Koç, 2016; Arslan, 2020; Plungian, 2001; Tosun & Vaid, 2018, in press; Willett, 1988). Given the substantial rise in MT usage and the importance of accurately conveying the source of past events, the ability to detect the accuracy of evidentiality translations in MT became crucial.

The study's findings revealed that both early and late bilinguals exhibited better error detection in firsthand sentences compared to non-firsthand sentences, but this difference was noticeable only when translations were from Turkish to English. In this context, late bilinguals were particularly adept at detecting machine translations. However, when the translation direction was from English to Turkish, the distinction between firsthand and non-firsthand sources vanished. Although firsthand sentences were somewhat more accurately detected than non-firsthand sentences, this difference did not reach statistical significance. These findings align with previous research, demonstrating that firsthand sources tend to be better remembered (Tosun et al., 2013) and translated more accurately (Tosun & Filipović, 2022).

Consistently, the study also highlighted the influence of AoA. Late bilingual speakers exhibited a greater awareness of the evidentiality distinction compared to their early bilingual counterparts (Arslan et al., 2015, 2017; Arslan & Bastiaanse, 2020; Karayayla, 2020; Schmid & Karayayla, 2020; Tosun et al., 2013; Tosun & Filipović, 2022). Most MT research has traditionally focused on examining proficiency levels rather than AoA. These studies have generally revealed that translators exhibit higher cognitive loads and lower acceptability compared to less proficient bilinguals (Carl et al., 2011; Castilho, 2016; Doherty, 2016; Daems et al., 2017; Ferreiraa et al., 2021; Guerberof Arenas et al., 2021; Hu et al., 2020; Kasperé et al., 2023; Moorkens, 2018; Stasimioti & Sosoni, 2021; Vardaro et al., 2019). In the current study, it was observed that late bilingual speakers exhibited a superior ability to detect errors in translations compared to their early bilingual counterparts. The late bilinguals in the sample also reported a higher overall proficiency level than the early bilinguals. These findings align with previous research on MT, underscoring the influence of proficiency levels and the consistency of these patterns in our study. It is important to highlight that the AoA, age of participants, and the language proficiency of the sample demonstrated a large overlap (where the late bilinguals were older and more proficient than the early bilinguals). This made it challenging to isolate the sole impact of AoA. Previous studies on age and MT usage (e.g., Kasperė et al., 2021) indicate that older individuals generally utilize MT less often compared to younger ones. However, intriguingly, in this study, late bilinguals (the older group) displayed more frequent MT usage than early bilinguals (the younger group), contrary to prior age-related findings. Future studies should prioritize investigating the distinct effects of AoA, age of participants, and language proficiency in machine translation (MT).

Conversely, the findings regarding the direction of translation yielded results that differed from prior research. In the present study, late bilinguals exhibited a greater proficiency in detecting the accuracy of Turkish-to-English translations compared to English-to-Turkish translations. This contrasted with the outcomes of Tosun and Filipović's study (2022), where bilingual speakers displayed more accurate translations when translating English sentences into Turkish. The inconsistency in results could be attributed to procedural differences between the two studies. In the current study, participants were tasked with determining whether the source sentence had been accurately translated into the target language. When the target language was English, participants encountered additional phrases like “it seemed” or “apparently,” influencing their decisions. These evidential phrases made the sources of information more conspicuous in the Turkish-to-English direction. In contrast, Tosun and Filipović's experiment required participants to produce the correct markers when translating between the two languages. The production of such phrases is relatively more challenging in English because the language does not mandate speakers to indicate the source of information. Consequently, their study found lower accuracy in translating from Turkish to English. Furthermore, the translation literature has explored the role of direction as a factor affecting translation accuracy (Ferreira & Schwieter, 2017; García et al., 2014). It was concluded that the advantage of forward (L1 to L2) or backward (L2 to L1) translations did not consistently yield robust results and was influenced by various factors, including experience and proficiency.

In terms of MT preference, the study revealed several noteworthy findings. The average accuracy in detecting MT errors related to evidentiality stood at 32%. In terms of preference for MT, the figures were as follows: 46% in medical settings, 53% in legal, 56% in academic, and 38% in daily settings. Participants reported using MT tools with a frequency of 21% and expressed a trust level of 65% in MT. The results demonstrated that, except in daily settings, the accuracy detection of non-firsthand sources significantly predicted preferences for MT. Intriguingly, the direction of translation (from Turkish to English or vice versa) had contrasting effects. Participants who more accurately detected non-firsthand translations from English to Turkish were less likely to prefer MT, while those who more accurately detected such translations from Turkish to English were more inclined to opt for MT. Similarly, in academic and daily contexts, those who reported lower trust in MT were more likely to prefer MT. Additionally, AoA emerged as a significant predictor, except in academic settings, with late bilingual speakers showing a greater preference for MT, even though they exhibited better accuracy in error detection compared to early bilinguals. These findings were consistent with prior research (e.g., García, 2010; Kasperé et al., 2021, 2023).

Summing up these findings, it becomes evident that despite the ability of bilinguals to spot errors in machine translations and their lack of trust in MT products, they still preferred MT. In response to an open-ended question regarding their preference for MT, participants commonly cited ease of access, cost-effectiveness, and convenience as the primary reasons. As Kasperé et al. () discussed, the bilingual speakers in this study indicated a preference for MT even when translations were only partially accurate because they valued the ability to convey their message quickly and conveniently. However, it is worth noting that trust in MT had a positive correlation with MT preference in legal settings, whereas trust in MT did not significantly predict MT preference in medical contexts. This discrepancy might be attributed to the critical importance of clear communication in legal settings, leading participants to be more cautious in their choice of MT (e.g., Asscher & Glikson, 2023). For future research, it is recommended to delve into the actual use of MT in vital contexts such as medical or legal, assessing both the accuracy of the translations and user satisfaction.

In summary, the study highlighted the influence of AoA on accuracy detection, particularly in aspects where a linguistic property is present grammatically in one language and lexically in the other. Alongside AoA, the accuracy of detecting non-firsthand sources and reported trust in MT emerged as significant predictors of MT preference, although the outcomes varied depending on the translation context. Furthermore, participants offered insights into their preference for MT, emphasizing factors like accessibility, cost-effectiveness, and convenience, even if the translations were only partially correct. For future research, exploring the attitudes and accuracy detection abilities of bilingual speakers with diverse backgrounds, including brokers (who are individuals, often children or adolescents, who facilitate communication between their family members and dominant language speakers), professional translators, and foreign language users, could provide valuable insights. In conclusion, the study underscores the multifaceted factors influencing preferences for MT, highlighting the significance of AoA, accuracy detection, and trust in MT across different translation contexts.

## Data Availability

The data supporting the findings of this study are available from the corresponding author on request.

## Footnotes

1 An a priori power analysis using the G*Power 3.1 computer program (Faul et al., 2009) demonstrated that a total of 30 people would be needed to detect the effects (f = 0.25) with 90% power (1−β) using a repeated measures (*r* = 0.5 among the conditions) ANOVA with a repeated factor, within-between interaction design with alpha at 0.05. While 30 participants provided the intended statistical power, a larger number of participants than originally planned were accessible, allowing for additional data collection.

2 The initial intent was to recruit an equal number of participants from each gender to best represent the population. However, during the data collection process, it became evident that only female participants were fully responsive.

3 The firsthand sources were not entered to the model as predictors due to collinearity issues. Because non-firsthand source is the critical distinction between Turkish and English typology, only these scores were entered to the model.
